# Ophthalmology

**Published:** 1893-01

**Authors:** Alvin A. Hubbell

**Affiliations:** Professor of Ophthalmology in the Medical Department of Niagara University, etc.


### Progress in Medical Science.

### OPHTHALMOLOGY.

By ALVIN A. HUBBELL, M. D.,
Professor of Ophthalmology in the Medical Department of Niagara University, etc.

### A NEW METHOD OF OPERATING IN PTOSIS.

BIRNBACHER (*Centralblatt f. Prak. Augenheilkunde*, May, 1892,) operates for ptosis in such a manner as to do the least possible injury to the skin, and, at the same time, bring about the occipito-frontalis muscle to act upon the tarsal cartilage directly by connective tissue bands passing from one to the other, so that the lid is raised without any rolling outwards or ectropion. He makes an incision through the skin along the whole length of the lid corresponding to the upper edge of the tarsal cartilage, which is then cleared and pierced by three double-needled, stout silk threads ; the middle one of these threads lies at the highest convexity of the tarsus, and the others at a distance of seven mm. from it to the right and left. The loops of thread are not knotted ; both ends of the middle stitch are carried vertically upward under the skin and emerge close together in the eyebrow, and the other stitches are treated similarly, but they are caused to incline somewhat away from the vertical, so that the points of emergence of the three are farther apart than the points of entrance. The three stitches are then tied over three iodoform pads, the guide as to the degree of tightness to which the stitches are drawn being that, on closure of the eyes, the upper and under lids can still touch. The skin wound is then closed with five fine silk threads. By this method, with the minimum of damage to the skin (for the points of emergence are hidden in the hair of the eyebrow), one obtains several short cicatricial bands, which have little tendency to stretch, uniting tarsus and frontalis muscle.

The threads are left in, under an antiseptic dressing, for twenty to twenty-five days, after which time one can feel quite distinctly the new bands referred to.—*Ophthalmic Review*, July, 1892.

---

### ACCIDENTAL VACCINIA OF THE EYELIDS.

MR. LATHAM THOMPSON, of Cardiff, at the July, 1892, meeting of the Ophthalmological Society of the United Kingdom, communicated notes of a case of a man, aged thirty-eight, whose left eyelids had become accidently inoculated. The lids were edematous and painful, and at the outer canthus the edges were involved in a purulent ulcer with indurated margins. There was great tenderness of the affected parts, and painful enlargement of the glands at the angle of the jaw and down the sterno-mastoid. The man's child had been vaccinated a short time previously, and his wife had been accidently inoculated therefrom. The man had suffered from slight marginal blepharitis, with excoriation at the outer canthus, and the inoculation had probably occurred at that point.

---

### EXPERIMENT BEARING UPON SYMPATHETIC OPHTHALMIA, WITH A CRITICAL REVIEW OF THE SUBJECT.

Dr. Randolph, of Baltimore, (*Archives of Ophthalmology*, July, 1892,) reports a very interesting case and experiment :

J. M., 41 years old, was injured in his sight, June 1, 1891. In about seven weeks afterward, the left eye, whose vision had been unusually acute, began to show signs of sympathy, viz., photophobia, lacrymation, and vision so impaired that he "could not see a horse a hundred yards away." August 13th, following the injury, vision of sympathizing eye equaled 10-200, that of the injured eye nothing. The injured eye was enucleated and endeavors made to grow experiment cultures from it on agar and in Esmarch tubes, material being taken from the anterior and posterior chambers. After proper methods and ten days' time, "not a sign of growth showed itself" on the culture media. Prof. Welch, Dr. Flexner, and Dr. Randolph examined specimens from the vitreous and anterior chamber of the enucleated eye, with the microscope, but discovered no organisms. Inoculation experiments were practised upon a rabbit's eye, but without inducing any symptoms of sympathetic ophthalmia in the animal. In short, the investigation was negative in its results throughout.

The author then reviews the experiments of Deutschmann and the theory of the pathogenesis of sympathetic ophthalmia which the latter has put forward. Gifford, of Omaha, Mazza, of Genoa, Leimbourg and Levy, of Strassburg, and Ohlmann and others, together with himself, have each failed to confirm Deutschmann's results. He, therefore, concludes that "it seems unreasonable to look upon the infection theory, as demonstrated by the experiments of Deutschmann, as anything, yet awhile, but an idea." It seems to him that his experiment in the laboratory, here referred to, is a most valuable test of the question, as to what *rôle* bacteria play in the production of the disease. Here is an undoubted case of sympathetic ophthalmia, and if bacteria in the injured eye gave rise to the sympathetic trouble, it is reasonable to suppose that the means to discover them would have been successful, and the experiment on the rabbit would, at least, have suggested something which might be regarded as positive evidence.

---

### PTERYGIUM.

E. FUCHS, of Vienna, (*Von Graefe's Arch. f. Oph.*, Vol. XXXVIII., Part II.,) publishes the results of his studies of pterygium, and declares that it is but a further development of pinguicula. After disposing of certain erroneous observations, and the theories that have been founded upon them and become current in the literature of the subject, he presents his own views, founded upon minute investigations, for which very ample material was at his disposal, together with careful and detailed clinical and histological descriptions. He concludes that a pterygium is a development of a pinguicula. The pinguicula, which ordinarily does not touch the limbus, gradually invades it and advances over it on to the cornea, causing a thickening at the limbus, which subsides somewhat steeply towards the cornea, and has a convex margin. In accordance with this mode of origin, the situation proper to pterygium corresponds with that of pingüicula, viz., the inner or the outer side of the ball, most frequently the inner, and somewhat below rather than above the horizontal meridian. The two disorders also belong to the same period of life. As the pinguicula continues to grow on the cornea, it loses its original characters, the yellow nodules disappearing and the advanced margin assuming a greyish, brawny look. Some parts of this margin advance more rapidly than others, giving a jagged or sinuous appearance. It is very intimately attached to the subjacent cornea, destroying, at this place, Bowman's membrane, and loosening the epithelium in advance of the margin. The adhesion of the pterygium to the corneal tissue varies considerably. Sometimes it penetrates the superficial layers in the form of loose, vascular, connective tissue, at other times it consists of a firm, hard tissue, adhering superficially to the undamaged Bowman's membrane. The former seems to indicate the progressive, the latter the stationary stage of the growth. Clinically, in the former, the margin is thick and brawny, with steep edge toward the cornea, while in the latter it is thin, tendon-like, and hardly raised above the level of the cornea.

It is evident that a pterygium is something more than a simple extension of a pinguicula, and its structure, moreover, does not resemble that of a pinguicula. It would appear that a pinguicula, near to the corneal margin, in some way modifies the lymph which here flows into the cornea, and thereby interferes with corneal nutrition at this point, and leads to a progressive destruction of Bowman's membrane and the subjacent layers of corneal substance. At the same time, a proliferation of connective tissue seems to be excited, so that while the destructive process proceeds around the apex of the growth, cicatrization follows behind it. As the growth of the pterygium proceeds, the characters which belong to the pinguicula disappear, gradually, and ultimately, when the pterygium has reached a considerable size, the destructive action upon the corneal tissue appears to be exhausted, the progress is arrested, and a certain amount of atrophy of the new tissue takes place.

Fuchs puts forward this hypothesis as one which needs support by further investigation, especially by the histological examination of pterygia in their early stages.

---

PROFESSIONAL HOMES are a real necessity according to the *Medical Age*, which says : The members of the medical profession in every city of 100,000 people should have their own home. They may be only able to rent a humble dwelling for such a purpose, but by all means rent until able to own, and there have a library, a reading and common sitting-room, a dining-room, and a kitchen. A fraternal spirit and union of hearts will there be knit together. Professional homes are promoters of peace and good-will, and from them is sure to spring the benevolent sentiment that encourages the strong to care for and not step aside to crush out the life of the weak and, perhaps, unfortunate. In the cultivation of this spirit and sentiment, strong men are made stronger, and their lives more lovable. Their angular points are smoothed down until they are so polished as to on all occasions reflect the nobleness of soul that is within them, while the weak, but deserving, are held up and encouraged in practical ways.—*St. Louis Medical Review.*

---

*The Medical and Surgical Observer* is the name of a new monthly magazine, published at Jackson, Tenn., and edited by Dr. Vandahurst Lynk.

